# Game-based intradialytic non-weight-bearing exercise training on gait speed and balance in older adults with diabetes: a single-blind randomized controlled trial

**DOI:** 10.1038/s41598-023-41290-3

**Published:** 2023-08-30

**Authors:** M. G. Finco, Bijan Najafi, He Zhou, Abdullah Hamad, Rania Ibrahim, Fadwa Al-Ali

**Affiliations:** 1 Interdisciplinary Consortium on Advanced Motion Performance (iCAMP), Michael E. DeBakey Department of Surgery, Baylor College of Medicine, Houston, TX USA; 2 Department of Nephrology, Fahad Bin Jassim Kidney Center, Hamad General Hospital, Doha, Qatar; 3 Shenzhen Mass Medical Co., Ltd., Shenzhen, China; 4 Shanghai Dengding BioAI Co., Ltd., Shanghai, China

**Keywords:** Geriatrics, Musculoskeletal system

## Abstract

Older adults with diabetes receiving hemodialysis have impaired gait speed and balance compared to the general population, which have been associated with increased risks of falls and mortality. This study evaluated the effectiveness of a game-based intradialytic exercise training program (iExergame) on improving gait speed and balance. This was a single-blind randomized controlled trial. The intervention group (IG) received iExergame training using real-time audiovisual feedback with wearable inertial sensors. The control group (CG) received conventional training without any technology. Both trainings were intradialytic, non-weight-bearing, and used ankle range of motion. Gait and balance parameters were collected at baseline and 4-week follow-up. Data from 70 adults (age 64.2 ± 9.0 years) were analyzed. Compared to the CG, the IG showed greater changes between baseline and 4-week follow-up in several parameters. Gait parameters included faster speeds and longer stride lengths, particularly during dual task walking (*p* < 0.050). Balance parameters included reductions in center of mass (*p* = 0.004), ankle (*p* < 0.001), and hip (*p* = 0.010) sways during semi-tandem stance, particularly in users of assistive devices. iExergame training could improve gait speed and balance in this population and might be an option to increase intradialytic exercise adherence while reducing burdens of exercise administration.

## Introduction

In older individuals with diabetes, low compliance with exercise and physical activity has been associated with foot complications (e.g., ulcers) and frailty, which can increase economic costs^1–4^. Older individuals with diabetes who receive hemodialysis (HD) may be at increased risks, since HD requires individuals to lay in a static position for up to four hours three times per week. Recent evidence suggests foot and ankle exercise programs can increase joint mobility and peripheral blood flow, which could help reduce risks of foot complications in individuals with diabetes receiving HD. However, low exercise compliance, potentially due to time availability, post-dialysis fatigue, and transportation to exercise programs, poses barriers to reducing risks of foot complications in this population^5,6^.

Delivering exercise during HD sessions, which is commonly referred to as intradialytic exercise, could help address these barriers to exercise^7^. However, implementation of intradialytic exercise has been slow, perhaps due to conflicting research recommendations and healthcare professional attitudes. Intradialytic exercise literature in older individuals with diabetes has shown inconsistent findings regarding benefits, and focused on aerobic and resistance training^8^. Healthcare professionals have reported lack of rehabilitation training, knowledge, and specialized exercise equipment as perceived barriers to implementing exercise in this population. Further, older individuals with diabetes receiving HD have reported disinterest as perceived barriers to exercise^9^. Thus, an intradialytic exercise intervention that is non-weight-bearing, coached, and gamified may help reduce foot complication risks, reduce healthcare professional labor, and increase exercise compliance in this population.

To help address limitations of conventional intradialytic exercise, we developed an interactive intradialytic exercise program (intradialytic-Exergame; iExergame) that is non-weight-bearing, coached, and gamified. Our previous research showed iExergame training significantly reduced depression in this population, and was well-received by participants^10^. In this study, we examined the influence of iExergame training on gait speed and postural control. Individuals with diabetes receiving HD have slower walking speeds and impaired postural control compared to the general population^11–13^, which have been associated with increased fall risks and mortality^14–18^. Specifically, this population has shown significantly more difficulty completing dual walking tasks (e.g. combination of cognitive and motor function)^19^, and impaired spatiotemporal gait parameters (e.g. shorter stride length, longer stride time^20,21^, and longer time in double-limb support) compared to age-matched controls. In summary, iExergame training could provide a non-weightbearing gamified solution to help improve gait speed and postural control in individuals with diabetes receiving HD, which could ultimately decrease fall risk and mortality.

This rationale informed our objective to evaluate the effect of a 4-week iExergame program on gait speed and postural control in older individuals with diabetes receiving HD. We hypothesized that compared to controls receiving conventional training without technology, older individuals receiving 4-week iExergame training would show: (1) increased gait speed, longer stride length, shorter stride time, and less time spent in double-limb support, as well as (2) decreased center of mass (COM), hip, and ankle postural sways.

## Methods

### Study design

This study was a parallel single-blind randomized control trial (ClinicalTrials.gov Identifier: NCT03076528, 10/03/2017) that investigated the influence of a novel non-weight-bearing intradialytic exercise training (iExergame) in older individuals with diabetes receiving hemodialysis. All procedures were performed in accordance with the Declaration of Helsinki. Additionally, our prior study describes the protocol of this study^22^. A computer-generated list (MATLAB software) randomly assigned participants in a 1:1 ratio to the intervention group (IG) or control group (CG). Only participants with complete data at baseline and 4-week follow-up were included in the final analysis.

For single-blinding, all participants were blinded to their group assignment, which was accomplished by not disclosing there was more than one group in this study. Specifically, participants were told the primary focus of this study was to ascertain the advantages of incorporating exercise into hemodialysis sessions, with potential improvements in balance and mobility highlighted. However, to ensure participant blinding, we intentionally did not reveal the specific exercise methodology—whether interactive via iExergame or a conventional, non-interactive program—at this stage. Post-group assignment, detailed information about the specific regimen was shared with the participants, strictly based on their group classification. The precise nature of the intervention, however, was only disclosed to participants upon the study's conclusion. Moreover, to further maintain the integrity of the blinding process, all care providers involved in the study were also kept uninformed about the group assignments.

The intervention group (IG) received coached iExergame training for 4 weeks, while the control group (CG) received equivalent training through a nurse-coached conventional exercise training for 4 weeks.

### Study population

**Figure 1:**
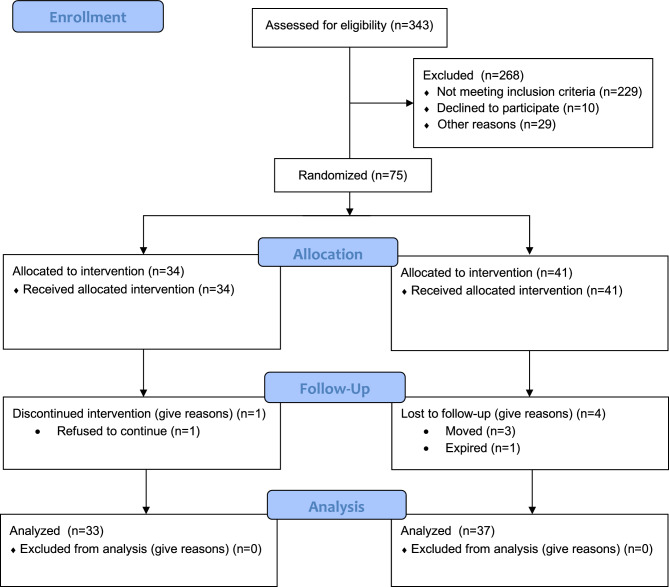
Consolidated standards of reporting trials (CONSORT) diagram for inclusion and exclusion of participants.

Participants were recruited from the Fahad Bin Jassim Kidney Center (Hamad Medical Corporation, Doha, Qatar), which is an outpatient hemodialysis clinic. To be included, participants had to be: 50 years of age or older, diagnosed with diabetes mellitus, currently receiving HD, and ambulatory. Participants were excluded if they had: current foot ulcers or infections, Charcot neuropathy, clinically significant medical or psychiatric conditions, major hearing/visual impairments, or experienced changes in psychotropic or sleep medications in the last 6 weeks. Additionally, participants were excluded if they were participating in any exercise training, or were unable to walk a distance of 15 m with or without an assistive device (e.g. cane or walker). Figure 1 depicts the number of participants assessed for eligibility, and those who were excluded or included. All participants signed a written consent approved by the Institutional Review Board at the Hamad Medical Corporation in Doha, Qatar. All methods and experimental protocols were carried out in accordance with the documents approved by the institutional review board. After providing informed consent, demographics were collected, which included: age, sex, mass, height, body-mass-index (BMI), duration of hemodialysis, use of assistive devices for walking, and daily number of prescription medications. The study protocol was approved by the Institutional Review Board at the Hamad Medical Corporation in Doha, Qatar. All participants signed a written informed consent.

### Sample size estimation

Our sample size was selected based on our previous study in the use of iExergame for older adults with diabetes^23^. An effect size of d = 0.891 was observed for benefit of a 4-week iExergame to improve balance compared to a group that did not participate in exercise. For the present study, we assumed a more conservative effect size of 0.80, alpha of 5%, and drop out of 20%, and an independent two-sample t-test. Therefore, recruiting 34 participants per group (with 27 participants per group to complete the program, considering 20% dropout) would generate a power of 82% or above to observe a significant difference between groups in response to iExergame intervention.

### Intervention group

The iExergame training used wearable sensors and a computer interface, which encouraged participants to utilize their full ankle range of motion in coronal (in/eversion), sagittal (dorsi/plantarflexion), and a combination of multiple (pro/supination) planes described in detail below. The IG completed the 4-week iExergame program under the non-weight-bearing condition during routine HD visits for 30-min per session (including breaks), totaling 12 sessions.

### iExergame

**Figure 2:**
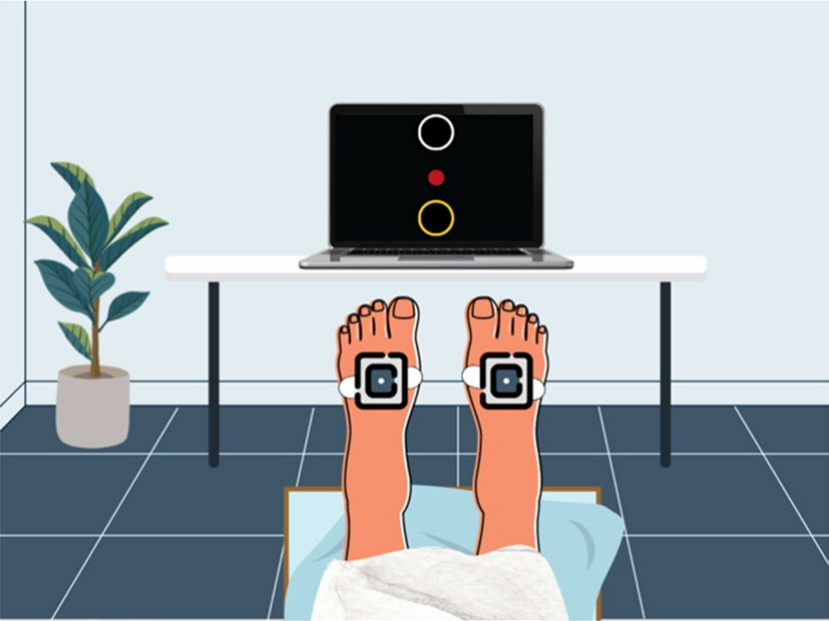
For the iExergame, an inertial sensor was attached on each foot, which converted ankle joint movement to computer cursor (red circle) movement.

Participants in the IG used the iExergame system, which consists of two wireless inertial sensors (LEGSys™, BioSensics, MA, USA), attached on the dorsal aspect of each foot by an elastic strap (Fig. 2). The inertial sensors contain a triaxial accelerometer (± 2 g) and triaxial gyroscope (± 2000 deg/s), enabling estimation of real-time foot motion using the Kalman filter and quaternion approach described in detail in our previous studies^22–24^. Sensor data were transmitted to the interactive interface (a computer laptop or smart tablet), at 100 Hz frequency for real-time feedback of foot motion.

The interactive interface screen includes a red square (defined as a cursor), one circle (defined as a home space), and one or more circles (defined as targets). The position of targets placed by the iExergame program depends on the type of desired exercise tasks. Participants were instructed to move the cursor from the home space to the desired target as fast as possible by rotating their ankle joint. Participants were equipped with a sensor on each foot during the exercises. The iExergame system was programmed to selectively read data from the pertinent sensor based on the type of exercise being performed—be it targeted for the right or left foot. Consequently, any movement from the contralateral foot had no influence on the cursor’s motion on the screen. Different gamification features, such as audiovisual rewards, were included to promote engagement and execution of each exercise task. For example, if the participant successfully brought the cursor from the home space to the desired target within 2 s, the target exploded and a bubble burst sound was played. Additionally, if an incorrect target was hit, the participant received audiovisual feedback to correct the error.

At the beginning of the iExergame program, each participant received brief instructions on how to perform the exercise. However, participants were not coached by a person during the exercise session, since the iExergame coached the participant via the interactive interface. If required, the program could be reinitiated and/or the complexity of exercise could be adjusted by the nurse who administered the exercise. For example, to complete one trial in the default program, the participant had to rotate their ankle approximately 20°. However, this range of motion could be changed depending on rigidity and frailty of the participants. Similarly, the timing of audiovisual rewards could be adjusted based on the participant’s baseline motor capacity.

**Figure 3:**
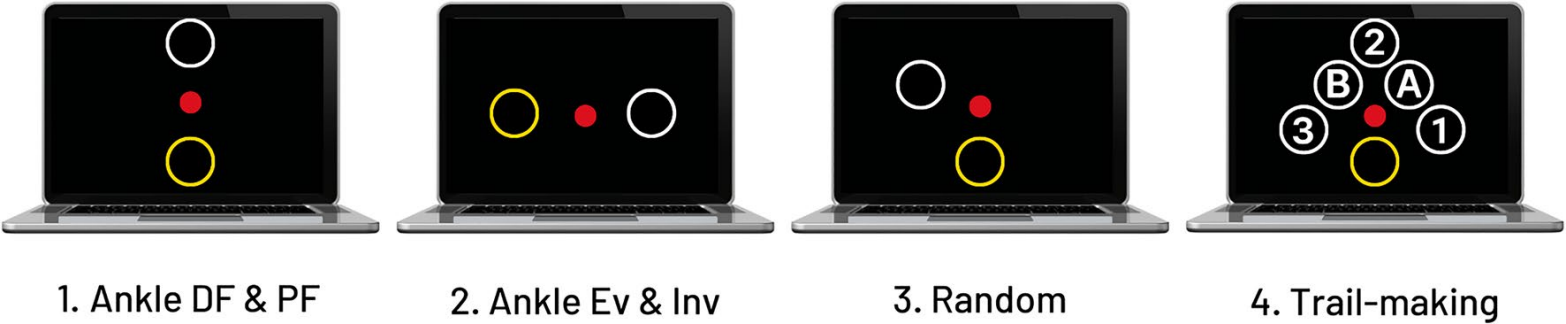
The iExergame program includes 4 different modules of exercises. Participants began with simpler exercises and progressed to more complex exercises that required more memory and cognition (from 1–4).

The complexity of exercises, similar to game levels, was adjusted based on participants’ performance in previous tasks. In this study, the nurse in charge made these adjustments. To guide them, a recommendation chart was provided, which referenced the scores from participants’ prior sessions. This helped tailor the exercise complexity or introduced more cognitively demanding exercises as appropriate. Four levels of complexity were included in the iExergame (Fig. 3), including:

1. Ankle dorsiflexion and plantarflexion exercise module: Participants needed to plantarflex their ankle joint to bring the cursor from the home space to the target, which was located on the upper part of screen. Then, when the target was hit, participants needed to dorsiflex their ankle joint to bring the cursor back to the home space.
2. Ankle inversion and eversion exercise module: Participants needed to bring the cursor from the home space to the target located on right or left part of the screen, by inverting or everting their ankle in medial and lateral directions.
3. Ankle rotation in random direction exercise module: Targets appeared on random locations of the screen, and participants needed to bring the cursor to the target as soon as they observed the target. These exercise tasks required a combination of ankle dorsiflexion, plantarflexion, inversion, and eversion. This module required more cognitive involvement compared to modules #1 and #2;
4. Trail-making exercise module: This module was similar to #3. However, 5 targets simultaneously appeared on the screen, labeled 1, 2, 3, A, and B. Participants needed to bring the cursor to the targets in the correct order, which was alternating numbers and letters. For example, first the target labeled ‘1’ was hit. Then, the cursor was brought back to home and in the next trial, the target labeled ‘A’ was hit. This module required a combination of memory, cognition, and motor function.

### Control group

Participants randomized into the CG completed a 4-week conventional exercise program during routine HD visits for 30-min per session (including breaks), totaling 12 sessions. In each session, they participated in a conventional foot-rotation training implemented by a nurse under the non-weight-bearing condition. Similar to the iExergame training, the conventional training involved the participant to move the foot through full range of motion in sagittal (dorsi/plantarflexion), coronal (in/eversion), and combined (pro/supination) planes. Therefore, the conventional training was the same as the iExergame training in visit frequency, duration of each visit, tasks, and intensity.

### Adherence monitoring

During routine hemodialysis sessions at the clinic (intradialytic exercise), all participants engaged in their respective exercise routines under careful nurse supervision. For those in the IG, the iExergame system provided comprehensive guidance via audio and visual cues on a computer screen. It set specific parameters, such as ankle range of motion and velocity, to achieve the desired exercise intensity. Meanwhile, the CG undertook a similar exercise routine under nurse supervision but without computer interaction. This meant that, unlike the IG, the CG did not benefit from the cognitive exercise components or the body awareness features facilitated by the iExergame system. At each session, the nurses diligently documented the exercise duration and assessed participants' adherence to the protocol.

### Outcome assessments

**Figure 4:**
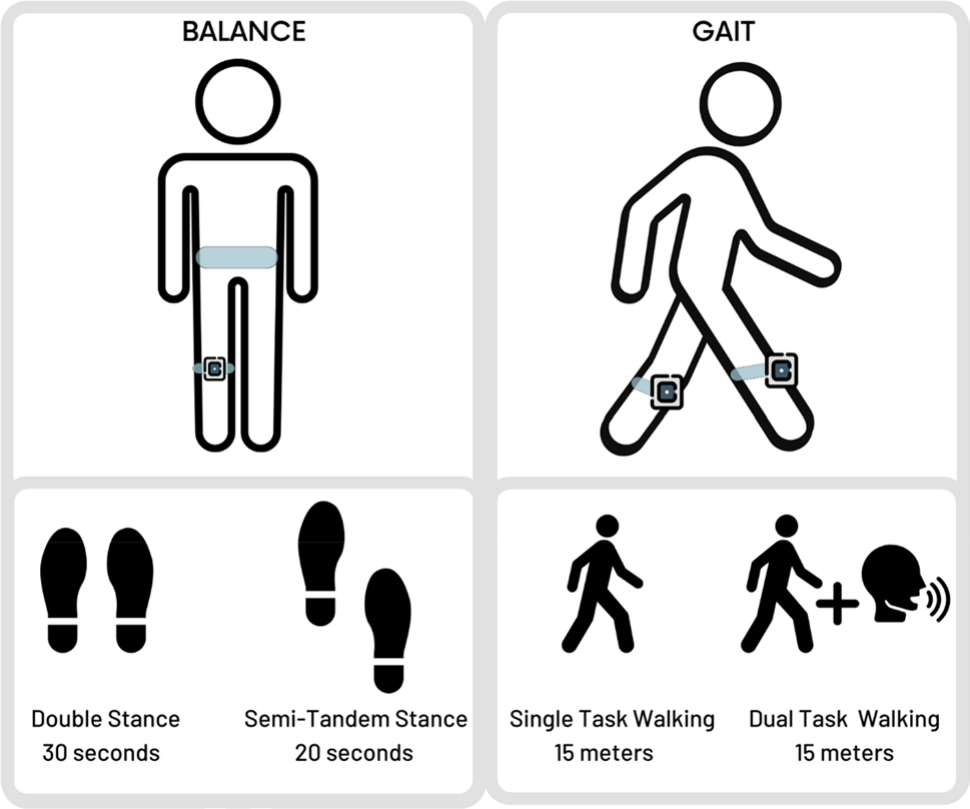
Balance and gait tasks performed using two wearable inertial sensors. For balance tasks, sensors were placed on the lower back and dominant shin. For gait tasks, sensors were placed on each shin.

Primary outcomes included changes between baseline and 4-week follow-up in gait, quantified by walking speed, and balance, quantified by center of mass (COM) postural sway (Fig. 4). Secondary outcomes included additional spatiotemporal parameters of gait including stride length, stride time, and double-limb support. Additional metrics of postural stability included ankle sway and hip sway. We also explored patient reported outcomes and user experience, which were reported in a previous study using this cohort^10^.

For gait tasks, the same two wearable sensors used in the iExergame were attached to the anterior aspects of left and right shanks to quantify spatiotemporal gait parameters. Participants were asked to walk at their habitual speed for 15 m without any distraction (Single Task Walking). Then, they repeated the test while counting backward from a random number in a loud voice (Dual Task Walking). One walking trial was collected per task (single and dual) due to participant fatigue, and the middle 5 strides of each trial were used in analysis. Gait speed (m/s), stride length (m), stride time (s), and double-limb support time (%), were calculated using validated algorithms^23–27^.

For balance tasks, one wearable sensor was attached to the anterior aspect of the dominant shank, and another wearable sensor was attached to the lower back. Balance parameters were quantified using BalanSens™ (BioSensics LLC, Newton, MA), which measures: ankle and hip motion in three dimensions, COM sway, and coordination between ankle and hip motion^28–30^. Balance tasks were performed during quiet standing (double stance task) and during standing with feet slightly moved anteroposterior (semi-tandem task). Both double stance and semi-tandem tasks were performed during eyes open and eyes closed. In the double-stance task, the participant stood in the upright position, keeping feet close together but not touching, with arms folded across the chest for 30 s. In the semi-tandem test, the participant stood with the dominant foot a half-foot behind the other, keeping feet close together but not touching, with arms folded across the chest for 20 s. Balance parameters, including center of mass sway (cm^2^), ankle sway (deg^2^), and hip sway (deg^2^), were calculated using a previous protocol and validated algorithms^28–30^.

### Statistical analysis

The Shapiro–Wilk test was used to assess the normality of the data. Between-group differences (IG vs. CG) were compared using independent t-tests. Analysis of covariance (ANCOVA) was used to compare the effect of training from baseline to follow-up measurements in each group. Since groups significantly differed in age, age was included as a covariate. A two-sided *p* ≤ 0.050 was statistically significant. Partial eta squared (*ηp*^*2*^) was used to determine effect size. Associations between the change in parameters between baseline and 4-week follow-up were evaluated using Pearson’s correlation. Generalized linear regression was used to assess group x time interactions. All statistical analyses were performed using IBM SPSS Statistics 25 (IBM, IL, USA).

## Results

Of the seventy-five recruited participants, data from 70 participants (33 CG; 37 IG) was analyzed (Fig. 1). The observed disparity in the number of participants allocated to each group (34 CG; 41 IG) stems from the nature of the randomization sequence employed. Our study initially set out with a sample size of 100 participants, but was concluded prematurely, leading to an unequal distribution of participants between the IG and the CG. This abrupt termination may have been a contributing factor in causing this resultant imbalance. As previously reported, both the IG and CG showed high adherence to the training regimen, with the IG participants expressing notable perception of benefit and ease of use with the iExergame^10^. However, one participant from the CG chose not to continue with the exercise program. Furthermore, five participants from the IG discontinued the exercise regimen due to various reasons: relocation (n = 3), passing away (n = 1), or hospital admission due to health complications unrelated to the study protocol (n = 1). No adverse events related to the iExergame training or conventional foot-rotation training were reported.

Demographic and clinical data are summarized in Table 1. The IG had significantly younger age (*p* = 0.021) and less individuals using assistive devices compared to the CG (*p* = 0.001). Therefore, all gait and balance results were adjusted by age. Gait and balance data are listed in Tables 2 and 3.

**Table 1:** Participant characteristics (mean ± standard deviation) in the CG and IG.

	Control (CG) (n = 33)	Intervention (IG) (n = 37)	*p*-value
Demographic characteristics			
Age, years	66.8 ± 10.3	61.8 ± 7.0	**0.021**
Females	19 (58%)	18 (49%)	0.455
Height, m	157.8 ± 10.6	157.1 ± 25.3	0.882
Mass, kg	82.4 ± 22.8	79.9 ± 16.4	0.593
BMI, kg/m^2^	33.1 ± 8.4	30.9 ± 6.4	0.213
Duration of hemodialysis, years	4.1 ± 4.0	4.7 ± 5.0	0.576
Using assistive device	23 (70%)	11 (30%)	**0.001**
Daily number of prescription medications	7 ± 3	8 ± 3	0.677

BMI, Body-mass-index; Significant difference between groups are indicated in bold.

**Table 2:** Gait parameters (mean ± standard deviation) at baseline and follow-up in the CG and IG.

	Control (CG, n = 33)				Intervention (IG, n = 37)				CG versus IG *p*-value	CG versus IG ηp^2^
	Baseline	Follow-up	% change	*p*-value	Baseline	Follow-up	% change	*p*-value		
Single Task Walking										
Gait speed, m/s	0.41 ± 0.20	0.40 ± 0.23	− 2.0	0.643	0.53 ± 0.19	0.56 ± 0.19	6.5	0.142	0.052	0.030
Stride length, m	0.61 ± 0.24	0.58 ± 0.27	− 5.2	0.180	0.75 ± 0.23	0.78 ± 0.20	3.5	0.333	**0.027**	0.038
Stride time, s	1.61 ± 0.33	1.63 ± 0.27	1.2	0.770	1.54 ± 0.32	1.44 ± 0.22	− 7.1	**0.035**	0.119	0.035
Double-limb support, %	44.0 ± 13.9	43.2 ± 12.6	− 1.7	0.572	34.2 ± 6.8	33.1 ± 6.0	− 3.1	0.250	0.248	0.000
Dual Task Walking										
Gait speed, m/s	0.41 ± 0.20	0.40 ± 0.20	− 3.7	0.612	0.50 ± 0.19	0.56 ± 0.18	13.1	**0.008**	**0.039**	0.071
Stride length, m	0.62 ± 0.24	0.58 ± 0.24	− 6.0	0.229	0.74 ± 0.25	0.79 ± 0.21	6.5	0.104	**0.037**	0.062
Stride time, s	1.65 ± 0.48	1.58 ± 0.27	− 4.1	0.359	1.56 ± 0.30	1.47 ± 0.24	− 5.5	0.069	0.759	0.001
Double-limb support, %	41.1 ± 12.2	39.9 ± 12.2	− 2.9	0.339	34.7 ± 8.7	33.6 ± 6.0	− 3.1	0.465	0.479	0.000

Results were adjusted by age. Percent changes with negative values indicate slower time or shorter steps at follow-up. Significant difference between groups are indicated in bold. Effect size was determined using partial eta squared (ηp^2^).

**Table 3:** Balance parameters (mean ± standard deviation) at baseline and follow-up in the CG and IG.

	Control (CG, n = 33)				Intervention (IG, n = 37)				CG vs IG *p*-value	CG vs IG ηp^2^
	Baseline	Follow-up	% change	*p*-value	Baseline	Follow-up	% change	*p*-value		
Double stance—eyes open										
COM sway, cm^2^	0.24 ± 0.19	0.37 ± 0.43	− 35.14	**0.024**	0.27 ± 0.28	0.27 ± 0.27	0.00	0.557	**0.010**	0.040
Ankle sway, deg^2^	3.27 ± 3.12	2.98 ± 2.08	9.73	0.518	3.06 ± 2.17	3.06 ± 2.25	0.00	0.197	0.694	0.003
Hip sway, deg^2^	3.39 ± 3.44	4.19 ± 4.29	− 19.09	0.982	3.15 ± 2.40	3.68 ± 3.34	− 14.40	0.052	0.364	0.001
Double stance—eyes closed										
COM sway, cm^2^	0.12 ± 0.11	0.17 ± 0.16	− 29.41	0.422	0.22 ± 0.23	0.20 ± 0.19	10.00	0.362	**0.040**	0.033
Ankle sway, deg^2^	2.06 ± 2.04	2.82 ± 2.31	− 26.95	0.612	2.52 ± 2.12	3.09 ± 2.98	− 18.44	0.267	0.597	0.001
Hip sway, deg^2^	2.04 ± 2.31	3.08 ± 3.52	− 33.77	**0.025**	2.66 ± 2.26	3.07 ± 3.04	− 13.36	0.354	0.111	0.009
Semi-tandem—eyes open										
COM sway, cm^2^	0.20 ± 0.17	0.45 ± 0.70	− 55.56	0.244	0.31 ± 0.33	0.22 ± 0.20	40.91	0.134	**0.003**	0.109
Ankle sway, deg^2^	3.26 ± 3.22	4.53 ± 4.47	− 28.04	0.074	3.45 ± 3.15	2.85 ± 2.38	21.05	0.086	**0.008**	0.050
Hip sway, deg^2^	2.93 ± 2.56	3.75 ± 3.18	− 21.87	0.873	4.21 ± 3.81	3.14 ± 2.76	34.08	0.427	**0.038**	0.061
Semi-tandem—eyes closed										
COM sway, cm^2^	0.17 ± 0.16	0.41 ± 0.51	− 58.56	0.792	0.24 ± 0.16	0.30 ± 0.29	− 20.00	0.909	0.074	0.044
Ankle sway, deg^2^	3.35 ± 3.60	5.28 ± 4.27	− 36.56	0.127	4.04 ± 2.86	3.16 ± 2.29	27.85	0.685	**0.002**	0.098
Hip sway, deg^2^	2.83 ± 2.93	4.94 ± 5.38	− 42.71	0.409	3.52 ± 2.42	3.56 ± 2.75	− 1.12	0.925	**0.050**	0.061

COM, center of mass; Results were adjusted by age. Significant differences are indicated in bold. Effect size was determined using partial eta squared (ηp^2^).

### Effects over time

For gait parameters, ANCOVA results in the CG did not show any significant changes between baseline and 4-week follow-up. However, the IG had a significantly slower stride time during single task walking (7% improvement, *p* = 0.035) and faster gait speed during dual task walking (13% improvement, *p* = 0.008).

**Figure 5:**
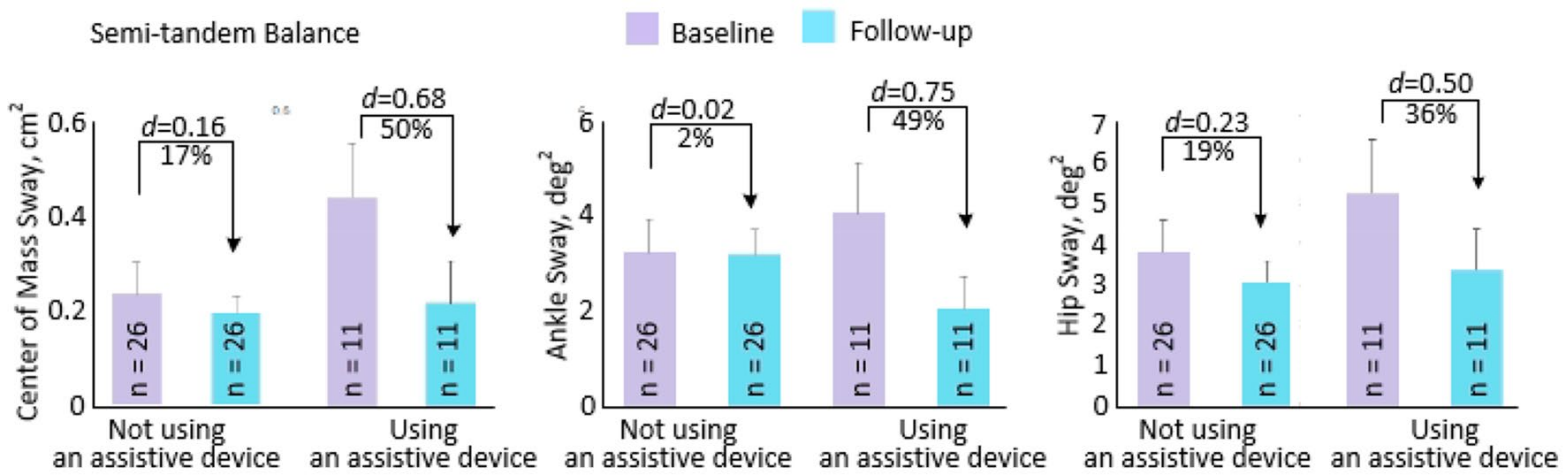
Center of mass, ankle, and hip sway at baseline (purple) and follow-up (blue) for Intervention Group participants only, grouped by whether or not they used an assistive device.

For balance parameters, ANCOVA results in the CG tended to show more sway in nearly all parameters between baseline and 4-week follow-up, while the IG tended to show less sway during the semi-tandem test. However, the only parameters that reached statistical significance between baseline and 4-week follow-up were increased COM (*p* = 0.024) and hip sway (*p* = 0.025) in the CG. The significant reduction in COM sway in the IG during the semi-tandem test (*p* = 0.003) is stratified by assistive device use in Fig. 5. While sway was reduced regardless of assistive device use, reductions in sway were more pronounced in participants who used assistive devices.

**Figure 6:**
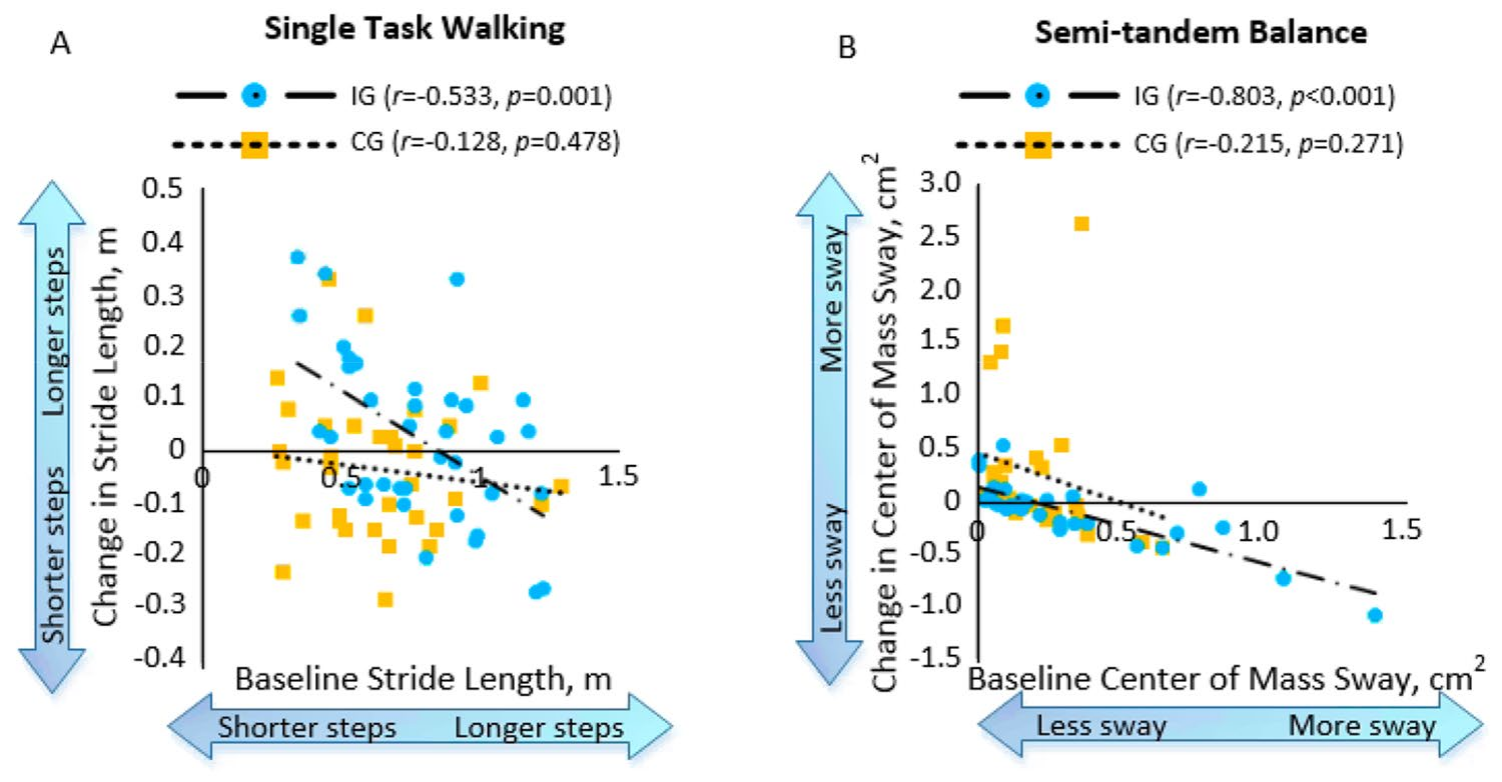
Significant negative correlations in the intervention group (IG) compared to the control group (CG). Changes in stride length between baseline and 4-week follow-up during single-task walking (**A**), and changes in center of mass sway baseline and 4-week follow-up during semi-tandem balance (**B**).

Correlations in the IG of gait and balance are depicted in Fig. 6. Figure 6A demonstrates the change of stride length between baseline and follow-up had a significant negative correlation with stride length at baseline during single task walking (*r* = − 0.533, *p* = 0.001). However, the correlation in the CG was weak (*r* = − 0.128, *p* = 0.478). Similarly, Fig. 6B demonstrates the change of COM sway between baseline and follow-up had a significant negative correlation with COM sway at baseline in the semi-tandem balance test (*r* = − 0.803, *p* < 0.001). However, the correlation in the CG was weak (*r* = − 0.215, *p* = 0.271).

### Effects between groups

At baseline, no differences between the CG and IG were observed for gait or balance. At 4-week follow-up, the IG had significantly longer stride lengths during single task walking (*p* = 0.027) compared to the CG. However, during dual task walking, the IG had longer stride lengths (*p* = 0.037), as well as faster gait speeds (*p* = 0.039) compared to the CG. The IG also had significantly less COM sway in the double-stance test during eyes open (*p* = 0.010) and eyes closed (*p* = 0.040) conditions. In the semi-tandem test, the IG group had significantly less COM sway during the eyes open (*p* = 0.003) condition, and significantly less ankle and hip (*p* = 0.010) sway during both eyes open (ankle: *p* = 0.008, hip: *p* = 0.038) and eyes closed (ankle: *p* = 0.002, hip: *p* = 0.050) conditions compared to the CG.

### Effects of group × time interactions

Gait parameters with significant group × time interactions included dual task gait speed (*p* = 0.022) and dual task stride length (*p* = 0.033). Balance parameters with significant group x time interactions were semi-tandem COM (*p* = 0.010) and hip sway (*p* = 0.046) during eyes open, and semi-tandem ankle sway during eyes closed (*p* = 0.009).

## Discussion

In this study, group x time interactions indicated that participants who received the iExergame training program had significantly faster gait speeds and longer stride lengths during dual task walking, as well as reduced postural sway, compared to participants who received conventional intradialytic exercise training. Additionally, of the participants who received iExergame training, those who used assistive devices demonstrated more improvements in postural sway from the iExergame training than those who did not use assistive devices during a semi-tandem balance test. Few studies have included older adults in this population. In this study, significant differences over 4 weeks were seen in older adults, whereas several studies with longer interventions found no significant differences in gait or balance. This may suggest older adults with iExergame training demonstrate more improvements in a shorter period of time than adults who are not older or do not use iExergame training. However, despite randomization, participants who received the iExergame training program were significantly younger than participants who received the conventional intradialytic exercise training, which may have influenced findings. More research is needed to assess the influence of age on the benefits of iExergame training.

Our previous study discussed participants’ self-reported perceived benefits of the iExergame training^10^. Specifically, the majority of participants reported: having fun while exercising, experiencing no problems or safety concerns, that the sensor feedback helped them learn exercises more quickly, and that the form and design was optimal. Further, several nurse and patient quotes, received since our previous publication, support these findings. A nurse reported, “all the patients are very happy with the exercises, appreciated the idea of performing exercises during their hemodialysis sessions, and mentioned that it made a difference even with their daily activities at home.” Another nurse reported, “This morning was the second session for participant 1. It was also perfect without any problems, completed exactly within 30 min…I can notice more improvement in the duration of exercise and the challenge of the patient trying to finish all the [exercises].” Finally, a participant reported, “I truly enjoyed performing [the exercises] during the hemodialysis session and I am very excited actually."

### Gait comparisons

Few studies in this population have found that exercise significantly increased walking speed^12^. However, our results demonstrated that with 4 weeks of iExergame training, older individuals receiving HD showed significant improvements in gait speed during dual-task walking, which is an essential component of motor control and balance. Individuals that received iExergame training also showed improvements in gait speed during single task walking, despite not showing significance. Mean gait speed from a recent systematic review across twenty-seven studies was 1.0 m/s and included few older adults. In contrast, this study of only older adults had mean gait speeds of 0.41–0.53 m/s at baseline. Therefore, individuals who are older adults or have slower gait speeds may demonstrate greater benefits from intradialytic exercise. The iExergame training could be studied in adults who are not older to determine if iExergame can provide the same benefits. In contrast to the IG, the CG experienced slight declines in gait speed in both single and dual-task walking conditions. This suggests the combined cognitive and motor control required to complete iExergame training may help improve cognitive-motor function in HD patients, and may help prevent declines in walking speed.

Stride lengths in participants included in this study (0.61–0.75 m at baseline) are comparable to stride lengths in previous research (0.60 m). Compared to controls, older individuals receiving iExergame training had significantly longer stride lengths during both single and dual-task walking conditions between baseline and 4-week follow-up. Shorter stride lengths and slower gait speeds have both been independently associated with higher fall risks in older individuals with stages 4 and 5 chronic kidney disease and end stage renal disease^17^. Therefore, this suggests that improvements in stride length with iExergame training may help reduce fall risks in this population. Further, significant group × time interactions of dual task gait speed (*p* = 0.022) and dual task stride length (*p* = 0.033) demonstrate the iExergame training was effective in these parameters.

### Balance comparisons

Participants tended to show impaired postural control compared to the general population, which aligns with previous findings^31^. However, few publications have investigated the relationship between exercise training and balance^32,33^. One study found that participants who had supervised group exercise showed more improved balance than those who had unsupervised home exercise, while others found technology-based exercise improved balance. In this study, participants who received conventional exercise training showed significantly more sway at 4-week follow-up compared to baseline in COM and hip sway during the double-stance task. However, no significant differences were observed in participants who received iExergame training. Despite not being statistically significant, individuals who received iExergame training typically showed less postural sway over 4 weeks, while those who received conventional exercise training typically showed more postural sway over 4 weeks. For between-group comparisons, individuals who received iExergame training showed significantly less sway at 4-week follow-up in nearly all parameters compared to individuals who received conventional exercise training. These findings over time and between groups suggest iExergame training may be able to help maintain balance in individuals receiving HD.

Individuals who received iExergame training that used assistive devices showed more pronounced reductions in sway during the semi-tandem test compared to those who did not use assistive devices. This finding suggests iExergame training may be more effective at enhancing balance in individuals who receive HD and use assistive devices. A recent meta-analysis provided evidence that wearable sensor training improved static balance in patient populations, including older adults and older individuals with peripheral neuropathy^34^. Older individuals who use assistive devices have been shown to have higher risks of falling compared to those who do not use assistive devices^35^. Therefore, iExergame training may help reduce fall risk in this population, but this relationship should be assessed in future studies. Additionally, significant group x time interactions during semi-tandem COM (*p* = 0.010) and hip sway (*p* = 0.046) during eyes open, as well as semi-tandem ankle sway during eyes closed (*p* = 0.009), demonstrating the iExergame was effective in these parameters.

### Correlations between baseline and follow-up

We observed a significant negative correlation between baseline and follow-up measurements in: (1) stride length during single task walking, and (2) COM sway during the semi-tandem balance task. This suggests individuals with shorter stride lengths and more COM postural sway (i.e. more impaired gait and balance) at baseline received more benefits from the iExergame training. Recent reviews have criticized light intensity exercises, short intervention periods, and disparate research findings in previous studies^8,36,37^. Despite the light intensity and relatively short 4-week intervention of the iExergame training, our results showed iExergame training could help increase gait speed and maintain postural sway in this population.

### Limitations and future research

Although randomization was done using a computer-generated list, the IG was significantly younger than the CG. To mitigate this, analyses were adjusted by age, but indirect age-associated differences (e.g. plasticity) may still have contributed to differences between groups. Additionally, the intervention period in this study was short compared to other training programs in HD patients. A longer intervention period may optimize motor learning and provide more benefits. The relationship between other factors, such as diet, risk of falling, daily physical activity, as well as clinician and participant attitudes towards iExergame training should be examined in future studies.

## Conclusions

This study suggests the coached non-weight-bearing iExergame training, which uses wearables and an interactive visual interface, can improve gait speed and maintain postural sway in older individuals with diabetes receiving HD. The iExergame can be performed while individuals receive HD in dialysis clinics, which could increase exercise adherence while reducing the labor of clinicians to administer exercise during HD. Future studies should determine if findings are generalizable and examine the long-term effects of iExergame training interventions.

## Data Availability

All data can be made available upon request by contacting the corresponding author.
